# An Economy Viewed as a Far-from-Equilibrium System from the Perspective of Algorithmic Information Theory

**DOI:** 10.3390/e20040228

**Published:** 2018-03-27

**Authors:** Sean Devine

**Affiliations:** School of Management, Victoria University of Wellington, PO Box 600, Wellington 6140, New Zealand; sean.devine@vuw.ac.nz

**Keywords:** emergence, economic complexity, economic order, non-equilibrium economics, energy and economic sustainability, Algorithmic Information Theory

## Abstract

This paper, using Algorithmic Information Theory (AIT), argues that once energy resources are considered, an economy, like an ecology, requires continuous energy to be sustained in a homeostatic state away from the decayed state of its (local) thermodynamic equilibrium. AIT identifies how economic actions and natural laws create an ordered economy through what is seen as computations on a real world Universal Turing Machine (UTM) that can be simulated to within a constant on a laboratory UTM. The shortest, appropriately coded, programme to do this defines the system’s information or algorithmic entropy. The computational behaviour of many generations of primitive economic agents can create a more ordered and advanced economy, able to be specified by a relatively short algorithm. The approach allows information flows to be tracked in real-world computational processes where instructions carried in stored energy create order while ejecting disorder. Selection processes implement the Maximum Power Principle while the economy trends towards Maximum Entropy Production, as tools amplify human labour and interconnections create energy efficiency. The approach provides insights into how an advanced economy is a more ordered economy, and tools to investigate the concerns of the Bioeconomists over long term economic survival.

## 1. Introduction

How can an economy the size of the United States of America support 325 million people, when such a population level would be impossible for simple hunter-gatherer communities ranging over the same territory? Algorithmic Information Theory (AIT) addresses this question by providing a framework to identify the thermodynamic requirements for a complex system such as an economy to be maintained far from thermodynamic equilibrium. From this perspective, a living system such as an economy, can be understood as far-from-equilibrium, real-world, computational system existing in a highly ordered set of states. The stored energy in the far-from-equilibrium real-world configuration carries the programme instructions that enact natural laws. The effect of these instructions, as captured by the second law of thermodynamics, is to drive the system to equilibrium where the energy that is released is dispersed as heat carried in the momentum bits. Because bits and energy are conserved in a reversible system, the energy per instruction bit in an isolated real world system aligns with the heat energy per bit produced. The number of bits in the shortest appropriately coded algorithm that specifies or generates the instantaneous state of the system, defines its algorithmic entropy.

In contrast to the idea of equilibrium used in neoclassical economics, energy considerations show that an ordered economy exists in a stable or homeostatic state, distant from its local thermodynamic equilibrium. This is the decayed state that emerges in the medium term when all energy sources are switched off and all economic life ceases (see Section 2.2). This perspective is developed to argue that a developed economy, as in the United States, is a highly ordered interconnected set of structures, having low algorithmic entropy, and which is able to be sustained distant from it local thermodynamic equilibrium, by using stored energy and ejecting high entropy waste.

A few researchers, such as [1], and also [2] have applied AIT to natural systems. In addition, Refs. [3,4] have defined the term “physical complexity” to refer to the environmental fit of a structure measured by the reduction in the length of its algorithmic description, given the information contained in its environment.

However, as used here, the algorithmic approach not only provides a set of tools to determine the thermodynamic requirements for a system to be maintained distant from equilibrium, but also provides insights into the trade-off between energy use and environmental costs as the economy grows, contributing to the debate between environmental economists [5,6,7] and the neoclassical economists [8,9].

The representative far–from-equilibrium living system that captures key thermodynamic principles is a system, initially of one bacterium, existing in a flow through of nutrients. The real-world instructions in the bacterial DNA access instructions stored in the chemical constituents of the nutrient flow, replicate bacteria until the their numbers reach the carrying capacity of the system. At the carrying capacity, the system is far-from-thermodynamic equilibrium, existing in an ordered homeostatic state, sustained by continuing computations that replace decayed bacteria and eject waste. As is discussed later, Algorithmic Information Theory provides a measure of the distance of such a system from thermodynamic equilibrium and, in so doing, identifies the entropy requirements to sustain the system. Similarly, the simplest economy of autonomous hunter-gatherer families reproduce (replicate) increasing their numbers to reach the carrying capacity. Additional complexity emerges, when replicating units cluster and become interdependent, forming tribes, firms and cities, driving the system further from equilibrium and, over many generations, creating a highly ordered first world economy.

Section 2 shows that the natural laws driving real-world behaviours are in effect computations on a real-world UTM. The number of bits in the shortest algorithm that exactly simulates the real-world computations defines the Kolmogorov complexity or algorithmic complexity of the system [10,11,12]. When an algorithm is appropriately coded, the number of bits in the generating algorithm defines the algorithmic entropy of the natural system [11,12]. The algorithmic entropy of an isolated real world system implies reversible computations. However, once reversibility is lost, tracking the bit inflows and outflows allows an economic system to be seen from a thermodynamic perspective as the thermodynamic entropy is related to the algorithmic entropy. The computational work that creates and sustains the economy, comes from the real-world programme instructions stored in the energy states of chemical resource species entering the system and in instructions stored in structures such as genes (see Section 2). Critically, know-how that exists in human brains as computational routines organises the information embodied in the external resources, levering off the genetic instructions to create more ordered structures. While such an economy becomes further from equilibrium, it becomes more dependent on resource inputs and must eject more waste. The “degree of order” of such a system is the difference in the number of bits between the algorithmic description of a low entropy or ordered far-from-equilibrium state, and the thermodynamic equilibrium state.

### 1.1. The Creation of Order by Economic Agents

The above principles provide an understanding of how information embodied in an economic system, drives growth and creates order, as seen in the complexity of its structures and interconnections. The more sophisticated the economy, the more information is stored in its structures, and the greater the resources needed to sustain it. While natural processes degrade a far-from-equilibrium economy, in order to regenerate the homeostatic state, energy must enter the system carrying the informational resources, and high entropy waste must be ejected from it. As illustrated in Table 1, there is a one to one correspondence between the information measured in bits embodied in the economy, and the energy flows in and out, as each computational bit must be carried by a unit of energy.

Resources→usefulenergy→informationasinstructions→order.

The decision-making unit at a particular level of an economy is denoted by the term “economic agent”. From a computational perspective, the agent maintains and grows the economy through instinctive routines such as “eat”, “reproduce”, and by cognitive processes which Hidalgo [13] calls “know-how”. These cognitive process, like a CPU, call routines in the natural world such as “burn wood” or “smelt iron” to alter the living environment. The primary behavioural driver in a simple economy of agents, such as hunter-gatherer communities is to reproduce (replicate) until the system reaches a homeostatic or stable state determined by the system’s carrying capacity. The system becomes further from equilibrium as the carrying capacity increases, for example when clothing or fire extend the viable range, or tools increase hunting effectiveness. This may happen by chance, or through cognitive processes that encapsulate know- how in better routines.

**Figure 1:**
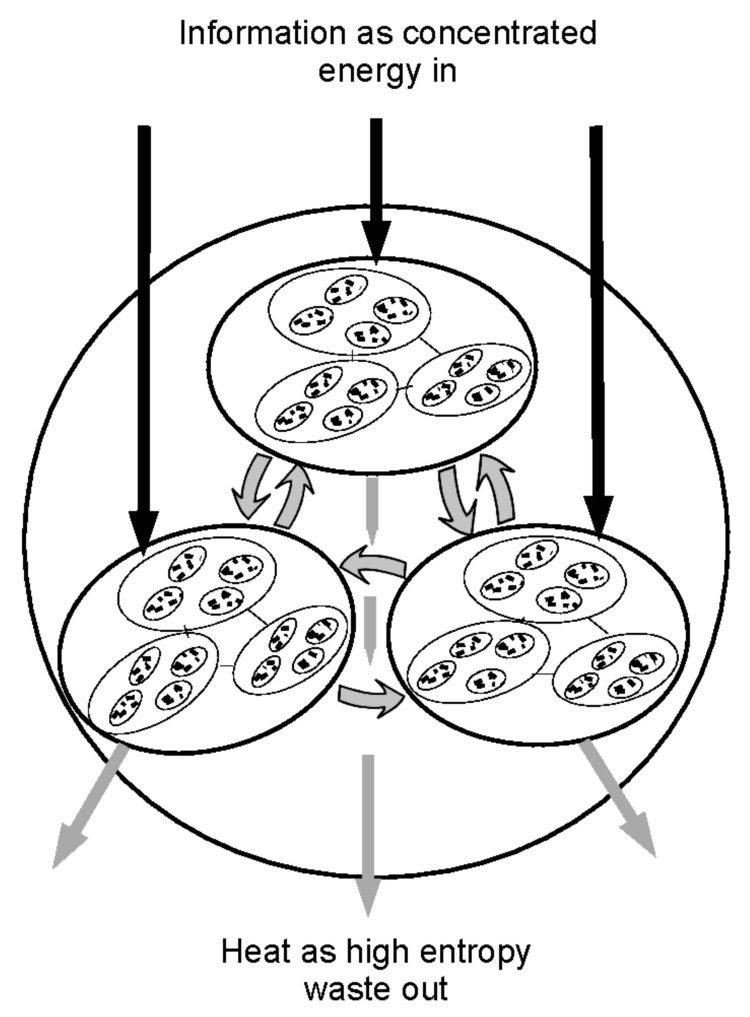
Resource and information flows in a nested and interconnected economy.

One can explore the emergence of a complex economy by a narrative that tracks the computational changes when a simple economy of autonomous, hunter-gatherer families evolves over many generations to form a large scale, more ordered and more connected economy. Ordered structures emerge when information efficiency increases, through developing networks, through trade, and through forming collectives such as tribes, firms, cities and nation states. This order is analogous to the order in a rich ecology, which because of interdependency and shared resources, is further from equilibrium. This is schematically shown in the hierarchical map of Figure 1. This shows how the routines and subroutines of an economy can be organised, enabling the system to be described by a shorter algorithm than would be the case if all the components were not interconnected. From a computational point of view, higher-level economic units call the computational subroutines that characterise the behaviour of the units at the next lower level of scale.

As is shown later, a more ordered economy not only uses resources more effectively, but can access wider range of resources than a less ordered one [14]. However, there is a cost. As more stored energy is required to sustain a first world economy, more waste must be ejected. The idea of order provides a framework to argue that the more ordered the economy, the further it is from equilibrium, and the greater the resources needed to sustain it. Furthermore, an ordered economy must be more effective in its energy use and its ability to eject disorder identified in high entropy waste and heat.

### 1.2. Notation

Here the phrase “more complex system” refers to a more highly organised system that is anything but random. On the other hand information theorist uses the phrase “more complex system” to mean a more disorganised system with little structure and which is described by a lengthy algorithm. There is further ambiguity as mathematicians define a more ordered system as one requiring fewer bits of information. While this is the definition used here, those who see a more ordered system as containing more information would find this counter intuitive. The “degree of order”, which measures the distance from equilibrium in bits, is closer to the intuitive meaning of information.

## 2. Information and Order

### 2.1. Algorithmic Specification of a System

The state of a far-from-equilibrium system, such as an economy at a particular instant, can in principle be represented by a string of digits or characters by a standard process. Whenever structure is observed in the system such as an economy, the string defining the state can be generated by a shorter algorithm than would otherwise be the case. A simple illustration is Conway’s Game of Life. It shows how a complex dynamic image of pixels can emerge on computer screen, by a simple algorithm using repeated rules [15]. Similarly, short algorithms generate the structures in an ordered economy relative to the algorithmic description of a completely disordered one.

As an example, consider the information content or algorithmic entropy measured in bits of an image of 1,000,000 random pixels on screen. If “0” represents a white pixel and “1” represents a black pixel, the string representing a random arrangement of pixels shows no structure and each of the million pixels needs to be specified separately. Such a random string is analogous to a string representing a random thermodynamic equilibrium configuration. The generating algorithm, *p* is of the form.

p=OUTPUT‘1001101…1100’.

As each pixel needs to be independently specified, the algorithm contains more than 1,000,000 bits. The information content, *H*, in bits is the length of the algorithm that generates the image, i.e.,

H=|p|=|OUTPUT|+|1001101…1100|+C.

Here the vertical lines |…| are used to denote the number of bits in the instruction bracketed between the lines. *C* is the simulation constant for the particular UTM running the algorithm.

However an image that shows order or structure can be compressed. The simplest ordered image is where every pixel is black (or white) and the compressed algorithm specifying the all black image just replicates the first “1”. In which case the algorithm is;

(1) p=REPLICATE‘1’amilliontimes.

The number of bits required to generate this image is close to;

(2) H=|p|=|REPLICATE|+|1|+|1,000,000|+C.

The OUTPUT and REPLICATE algorithms are both short and of similar size, i.e., |OUTPUT|≈|REPLICATE|. As 1,000,000 can be specified by $log_{2}\left(1,000,000\right)=20$ bits, the simple black image can be generated by an algorithm with a little more than 20 bits. The difference between the 1,000,000 bits needed to produce the random image (which would correspond to an equilibrium configuration for a completely decayed living system), and the black ordered image is 999,980 bits. This difference is the degree of order. Order is rare, as the overwhelming majority of the arrangements or configurations of an image of 1,000,000 pixels will show negligible pattern or structure. However, there is no certainty that a particular algorithm is the shortest possible.

If one also imagines that heat can enter the system and randomly degrade black pixels to white pixels and vice versa, instructions must enter the system to regenerate the original image. This is analogous to the way a living system, like an economy, needs useful energy containing programme instructions to reverse decay. A simple economy, initially of autonomous agents operating under simple instructions, generates complex structures (similar to the game of life) over many generations. In this case, the emerging structures evolve in real space rather than image space. However, to avoid decay under the second law of thermodynamics, this system must continually regenerate by accessing information carried in the stored energy by doing the equivalent of computational work, creating heat which is ejected with waste.

The degree of order, and the number of bits to specify an ordered system are in principle a measurable characteristic of a system and is closely related to Kolmogorov’s randomness deficiency [12]. The randomness deficiency may be somewhat shorter than the degree of order, as the algorithm that specifies the ordered state takes the length of the specification of the random state as an input.

#### The Algorithmic Entropy

The natural laws that drive real-world behaviour are in effect computations on a universal computer known as a Universal Turing Machine (UTM). The energy states of chemical species with their rules of interactions are the equivalent of gates in a conventional computer [16,17]. However, as one UTM can simulate another to within a constant, a laboratory reference UTM, using binary coding, can in principle simulate the natural computations implementing physical laws in the real-world. The number of bits in the shortest algorithmic description on the laboratory UTM that exactly defines a microstate of a system and halts, is known as its Kolmogorov complexity [10], or its algorithmic complexity [11,12]. However, as real-world computations are processed instantaneously, instantaneous or self-delimiting instructions are needed for the laboratory simulation [11,12,18]. In which case, the length of the programme is called the algorithmic entropy, as this measure in bits is closely related to the Shannon and thermodynamic entropies [16,19,20].

Only the entropy difference between two states has physical significance. The difference in algorithmic entropy between two states is independent of the UTM, as the contribution of any simulation instructions cancel. This allows the laboratory UTM to become a ruler to measure information distances in convenient units (i.e., bits) between two real-world configurations.

Because an isolated real-world system is reversible before any interaction with the environment, either a reversible UTM is needed to specify the system, or an irreversible computation must record the history states that provide reversibility information [16]). The reversible programme that generates a particular state will be the shortest possible specification as, with reversibility, there can only be one trajectory to a particular state if one ignores the backward path from the future. However, the algorithmic entropy measure, the number of bits defining a particular configuration, is conceptually different than the traditional entropies which assign the entropy to a macrostate consisting of a myriad of microstates. For example the Shannon information, $H_{sh}$, is a property of all the available states, i.e., $H_{sh}=log_{2}\left(available\;states\right)$.

However the algorithmic entropy of each individual microstate in a thermodynamic macrostate is known as the provisional entropy [21] and corresponds to Kolmogov’s algorithmic minimum sufficient statistic [12]. The provisional entropy consists of two terms. The first picks out the particular configuration in the thermodynamic macrostate, and the second defines the properties of the macrostate [21]. When there are Ω states in the macrostate, the Shannon entropy is $log_{2}\Omega$, while $log_{2}\Omega$ bits are required to pick out a particular configuration in the algorithmic approach. As a consequence, the provisional entropy is;

$$H_{sh}+H\left(macrostate\;specification\right).$$

The first term expresses the randomness of the state of interest, while the second its regularity. As the second term, which aligns with the “Effective Complexity” [22], is often negligible relative to the Shannon term, the provisional entropy may well be the same as the Shannon measure for all strings [21]. As Appendix A.1 outlines, when a reversible algorithm *p* generates a microstate in an equilibrium macrostate from within the system, |p| also $=H_{sh}$, allowing for residual information to maintain reversibility. While the provisional entropy is a measure external to the system, the requirements that appear in the definition of the macrostate, such as the total energy of the system, are implicit in the initial state and the residual information needed to maintain reversibility.

The algorithmic approach only selects the set of microstates satisfying the constraints in the term H(macrostatespecification), such as the energy requirements of the macrostate. The Maximum Entropy formulation [23] uses these same constraints in the Lagrangian multiplier term. This ensures that a microstate selected randomly from the set of allowable microstates in the algorithmic approach, will follow the Maximum Entropy distribution.

AIT relationships, such as mutual information and conditional information, take a similar form to the equivalent relationship for the Shannon entropy [11,19,21].

As is discussed in more detail later, in principle, a modern economy can be seen as the endpoint of computational processes that generates an advanced economy from a primitive one. The generating process can be followed by tracking information flows into and out of the system bits. As the algorithmic entropy is a function of state, one can either specify the state of a system with the shortest algorithm, or track the flow of bits in and out of an evolving system. This is similar to the way one might track the final state of a tree from its DNA instructions in the seed cell and the information flows embodied in the resource inputs and waste outputs. Ayres [24] Equation (10), articulates a similar argument for the Shannon entropy of a system consisting of *n* compartments. He shows that the Shannon entropy is equal to the entropy (which here would be the programme bits) in, less the entropy out, plus the entropy captured in the structure, i.e., the net flow of bits is identical to the number of bits in the length of the shortest algorithm that halts after generating a snapshot of the system’s configuration at a particular instant [17].

### 2.2. The Economy as a Far-from-Equilibrium System

A simple example of one bacterium immersed in a flow of nutrients shows how a far-from-, ordered living system can emerge. Given one bacterium, the population of bacteria will replicate by harnessing the energy in the nutrients, until the number of bacteria reach the system’s carrying capacity. This is determined by the efficiency of the process, and the rate of flow through of nutrients. At the carrying capacity *N*, the bacterial system can be specified by a short algorithm (embodied in the bacterial DNA) consisting of a routine of the form,

FromNutrientsREPLICATEbacteriumNtimes.

This is short and is analogous to Equation (1). The algorithm’s length consists of the length of the replicating algorithm (in the DNA) plus $log_{2}N$ [20]. Replication processes are at the heart of far-from-equilibrium living systems by creating an attractor in the state space. However, if the nutrient flow is stopped, the system will revert to a disordered local equilibrium of randomly distributed chemicals that can only be specified by an horrifically long algorithm.

The creation of order by replication can be seen as a computational process that accesses programme information in the nutrients in order to reproduce, while ejecting waste and heat. Humans are a collection of replicated cells generated by their DNA. A human family is a higher order replicating collective, which is even further from equilibrium. A collection of families that form a tribe is even more ordered and more distant from equilibrium, as the carrying capacity is higher. An economy is an ordered far-from-equilibrium collection of replicating subsystems that show identifiable pattern, structure and organisation, and which is simpler to describe than a less ordered one. The simplest economy of peasant farmers or hunter gatherers is sustained far-from-equilibrium, primarily because the participants consume food as the fuel to maintain their existence and to reproduce to replace the participants that die. In order for the most primitive economy to survive, disorder, as heat and human waste in the form of heat, sweat, carbon dioxide, water, excrement and corpses must be ejected.

The traditional understanding is that an economy reaches its optimum capability at an economic equilibrium where economic forces balance. However, once energy considerations are taken into account, what might be considered a neoclassical equilibrium is instead seen to be a homeostatic state distant from its thermodynamic equilibrium. Significant deviations from a homeostatic, stable configuration are path dependent and are usually irreversible. Although in the short term, forces might return the system to the homeostatic state. For example, if some agents die through drought in a hunter gatherer economy, when the rains return the number of agents could well return to the pre-drought situation. In an economy, when oil prices rise and then fall, the far-from-equilibrium economy does not necessarily return to the previous stable state, but may move to a new situation as the economy adjusts to alternative fuels or becomes more fuel efficient. Nevertheless, economic agents produce, consume and trade, make investments and strategic choices, exactly as expected, from a neoclassical or evolutionary economic perspective. Such an economy can follow a growth trajectory when more order is being created from computational processes driven by useful or stored energy. This is because the homeostatic state is an attractor maintained by the in-flow of resources and the out-flow of waste. Once all energy resources are turned off, death and decay processes implementing the second law of thermodynamics drive the system to its local equilibrium whether it be a human, an ecology, or an economy. The result is disordered arrangement chemical species with heat uniformly dispersed devoid of economic life.

A more sophisticated economy, with its dependence on fossil fuels and minerals, has more serious issues to face. When the waste, such as carbon dioxide, cannot be adequately ejected, disorder accumulates driving the system from its highly ordered state.

#### Energy to Store Information

AIT provides the mathematical tools to track how this order emerges by tracking the useful energy, that stores the programme bits, flowing into and out of a system. Information (as used here) is physical [25], and energy is required to store and process information in computer memory, in the human brain, or in the states of atoms and molecules. If the computational device is at temperature *T*, the extra energy to define one bit must be proportional to $k_{B}T$, to distinguish the extra energy state from the back ground average energy of $k_{B}T$ per bit, where $k_{B}$ is Boltzmann’s constant. Landauer [26] quantifies this by showing that when one bit is erased from a system, the energy cost is $k_{B}Tln2$ Joules per bit. From a computational point of view, as living systems are reversible at the level of the microstate, the original microstate can be restored by a reverse flow of $k_{B}Tln2$ Joules per bit. At the macro-level, dissipation for an irreversible process would make this heat flow greater. The validity of Landauer’s principle has been discussed by Devine [20]. Table 2 correlates the computations implementing natural processes with the corresponding natural processes. This has the important implication that energy flows in a non equilibrium system can be related to bit flows, and hence the requirements to maintain such a system away from equilibrium, i.e.,

- To reiterate, the algorithmic information flows that shift one real-world configuration to another are independent of the UTM used to map the process.
- It follows from Landauer’s argument above that, not only are bit (or any equivalent information unit) flows UTM independent, but for reversible systems, the energy associated with each bit is UTM independent and fixed at $k_{B}ln2T$ for a UTM operating at temperature T. The implication is that bits stored as programme instructions carry the same energy as bits that are ultimately stored as heat in the momentum states of the system, i.e., the energy that initially stores the instruction bits that ignite hydrogen and oxygen to from water at a higher temperature become momentum bits carrying the energy that is released. If *N* momentum bits need to be erased by computations in the external environment to return the system to the initial temperature, the heat removed is $Q=Nk_{B}Tln2$, as erasure is reversible over the short term. As both energy and bits are conserved in the reversible system, this must be the same as the energy released in the chemical reaction.

Indeed, Lloyd [27] in a Nature article argues the thermodynamic entropy stored in a volume of material containing *N* bits as $Nk_{B}ln2$ (see also [28]).

### 2.3. The Know-How Contribution to Driving an Ordered Economic System

In practice it would be impossible to track the binary algorithms that might generate or specify an economic system. Important routines are in the know-how that shifts the economy to another state by calling programmes embodied in the molecules in the natural world. Hidalgo [13] refers to the quantity of know-how embodied in human cognitive processes as “personbytes”. The human know-how contribution that manages the real-world computations can be expressed as routines analogous to the routines of Evolutionary Economics [29], or the heterogeneous expectations of the agents in the simulation of the stock market [30]. Other routines might be related to learning such as “imitate other agent’s behaviour”. However, deeply embedded human routines, common to all agents, such as routines that involve digesting food, extending muscles or fighting disease, can be ignored.

In principle, the know-how routines can be represented by equivalent binary programmes that capture human action. The energy carrying the know-how routines is insignificant and is probably indiscernible from the background routines sustaining a human being. However, it is not the aim here to investigate the processes whereby humans arise at these routines, or to specify them in detail, but to recognise that in principle they are part of the economy’s computational processes, and the discovery of routines embodied in know-how are part of the adaptation processes of human agents.

The information approach, that encapsulates agent behaviour in computational routines, provides particular insights into such questions as resource dependence, sustainability, and the drivers of an effective economy. From the perspective here coal or oil are inefficient energy sources compared with solar, because more energy must enter the system and more waste must leave, even though the energy difference is independent of the energy source.

The next section uses the algorithmic approach to investigate the trade-off between economic growth and environmental and energy costs.

## 3. The Trade-Off between Resource Costs and Economic Growth

A critical strategic issue is whether economic growth can continue indefinitely or whether there are natural limits. Georgescu-Roegen and the Bioeconomics’ school, believe that economies are constrained by the “entropy law” [5,6]. This so called law captures the point that all life, including economic life, operates as far-a from-equilibrium thermodynamic system. As a consequence, Georgescu-Roegen claims that the economy is constrained by the need to access “low entropy” and eject high entropy waste to maintain stability. On the other hand, Refs. [8,9,31,32], argue that as resources are depleted, the substitution of capital for labour can lead to technological improvements which compensate for resources used. Daly [33] reiterates Georgescu-Roegen [34] view that Solow and Stiglitz’ production function argument is a conjuring trick and an inevitable consequence of its homogenous form [33], thus avoiding the problem, or shifting it too far into the future.

However, Georgescu-Roegen has poorly articulated a crucial issue, borrowing his argument from Schrödinger’s book “What is life?” [35]. Shrödinger later apologised for oversimplifying by using the term “low entropy”, pointing out that it was free energy, not low entropy, that drives life. The ox, as a far-from-equilibrium system, does not directly import low entropy, but is sustained by accessing free energy from grass as fuel, and excreting waste containing heat. Georgescu-Roegen [36] further lost credibility by introducing a problematic 4th Law of Thermodynamics to argue material resources also degrade irreversibility [37].

While Georgescu-Roegen sees economic de-growth as ultimately necessary Daly [38], offers the steady state economy as an alternative to de-growth and later the quasi steady-state economy, similar to what is here called the homeostatic state [6,39]. Since the work of the Bioeconomics school, Ayres [7] (p. 39) has provided a more robust approach to the concerns of Georgescu-Roegen. Ayres [7] identifies confusions over terms such as entropy, low entropy, negative entropy, order, disorder, complexity and information; confusions that inhibit the conversation (see also [40,41]). Importantly, Ayres [24] draws attention to the thermodynamic entropy equivalent of one bit of information. If one bit of uncertainty is added or removed, the thermodynamic entropy change is $=k_{B}ln2$.

Ayres [7] argues that the Shannon measure of uncertainty is not the critical information measure. Instead he introduces the concept of D-information to specify the reduction in uncertainty when an observer gains more evidence about the state of the system indicating that it belongs to a subset of the original possibilities. If the subset is more ordered, this measure aligns with the degree of order used here. Ayres [7] also defines the term “physical information” for information that is stored in the energy states of real-world structures. Physical information provides the available energy, or exergy, corresponding to a generalised Gibb’s free energy, able to do useful work [5,42]. From the algorithmic perspective, Ayres’ physical information is the programme information that carries the computational instructions driving the system’s trajectory, separating order from the waste, ultimately ejecting of high entropy waste as heat and degraded chemical species. For example an isolated system of hydrogen and oxygen is distant from equilibrium. However, once ignition takes place, the physical information, as the computational bits stored in the energy states, is released forming water and heat, and in so doing, drives the system to equilibrium. The gain in bits in moving the system to equilibrium corresponds to D-information, as the uncertainty has increased by the number of bits added. Exergy only creates order when waste and heat are ejected from the system.

Ayres uses another term, “Survival Relevant Information”, denoted by SR, to refer to physical information that is relevant to evolutionary selection processes. SR information is the computational information stored as useful energy in atoms or molecules. While Ayres seems to restrict this concept to just the structural information representing capability in genes or, in the economic case, know-how, the algorithmic approach would also identify the grass that maintains the ox as important. All SR information, good or bad, is embodied in the programmes that impact on say an economic system. It includes the know-how, as well as the information and the real-world programmes that the know-how organises to bring about change.

Ayres [37] has a more moderate position than the Bioeconomists. While agreeing that resource flows are limited, he suggests solar energy is sufficient in principle to meet energy needs into the foreseeable future. With this view it then becomes possible to increase energy to compensate (at least to some extent) for decreasing mineral resources. Because in the long terms resources may be scarce, ultimately, as Ayres [37] notes, the future may need to recycle and mine the partially degraded waste from its past.

Critically, Ayres [7] has identified that, from an entropy perspective, manufacturing processes have become more efficient. He observed that in the 19th century economic value was added by harnessing information in chemical transformations by inefficient processes. A century later value added was mainly through harnessing information through more efficient morphological transformation. More recently, value tends to be added through symbolic information, effectively in the form of software. Ayres and Warr [43] then suggest that since 1970, as economic growth has been higher than the inputs of capital, labour and work, entropy as symbolic information is becoming more important. Seeing information in terms of programme bits gives a better understanding of Ayres’ insights. The order or information in a manual typewriter is morphological, whereas a PC embodies order mainly in the software and, as a tool, can achieve far more than the typewriter.

While technological advances can increase efficiency of resource use, and recycle waste with residual stored energy, new technologies increase demand for useful energy in the first instance and require learning to be used effectively. AIT complements Ayres’ approach and can provide insights into the trade-off between energy use and environmental costs to sustain a far-from–equilibrium economy. Also it provides a useful tool with which to engage with more traditional views. The rise of atmospheric carbon dioxide that disturbs the balance of the economy is seen as a failure to effectively eliminate waste. Nevertheless, a key point, is that order can continue to increase, even in the quasi steady-state system with little GDP growth. Greater awareness of the operational constraints of an economy will help societies to maintain their way of life.

## 4. Alternative Approaches to Complexity and Information

### 4.1. Control Information

Corning and Kline express concerns over the loose terminology in discussions centred around the Bioeconomics’ school [40,44,45]. They argue that the Shannon idea of information as uncertainty is blind to any functional basis the information might have. Instead they introduce the idea of “Control Information”, which from a cybernetic perspective is; “The capacity (know-how) to control the acquisition, disposition and utilization of matter/energy in purposive (teleonomic) processes” [44,45]. In practice they take control information, $I_{CI}$, as

$$I_{CI}\propto \left(lnA_{use}-lnA_{cost}\right),$$

where $lnA_{use}$ refers to the natural logarithm of the available useful stored energy, while $lnA_{cost}$ is the natural logarithm of the energy expended to harness the useful energy. From this perspective, driving a car has high energy returns, but with little cost identified as the energy needed for humans to control the process. Riding a bicycle has a similar low cost, but much lower energy returns as $I_{CI}$ is lower.

The AIT measure of information avoids the problems of information as uncertainty, as it is the number of programme bits that execute a function in the real-world. The AIT equivalent of $I_{CI}$, is what here would be called the control information in bits specified by $H_{CI}$. This is the combination of the human know-how and the real-world subroutines that achieve a specific purpose (such as smelt iron), less the cost in doing so. The appropriate proportionality constant to turn energy to information is $1/(k_{B}ln2T)$ bits per Joule. Hence the algorithmic equivalent of control information would be,

$$H_{CI}=H_{use}-H_{cost}.$$

However, the concerns in the current paper are not about energy efficiency or profitability, but the long term sustainability of the economic processes. An economy is less under threat from resource constraints when bicycles are used for transport compared with motor vehicles, even though the control information or information profitability of motor transport is higher.

### 4.2. Energy Flows in Low Entropy Systems

Refs. [41,46] has developed a big picture view of the common features of complex systems ranging from the grand scale of the cosmos to societal complexity. He notes that, as complex systems are islands of low entropy, entropy in the environment must be increasing. Chaisson suggests that the idea of information is of little use in quantifying complexity in the general case. Instead he sees entropy decreases in complex systems as dependent on energy flows. Human society with its machines are among the most energy laden systems known. Consequently, he defines the measure “energy rate density” as the measure of complexity. This is the amount of (free) energy available for work that passes through a system in unit time and unit mass [41,46]. For example, the energy rate density ranges from 4 Joules/sec/kg for a hunter-gatherer society to 200 Joule/sec/kg for a modern society. The cultural evolution of sophisticated gadgets can be traced by the rise in energy rate density. Chaissson notes that the energy rate density rises sharply for complex system and then tapers off. This would be seen here as due to a rapid rise until the carrying capacity of a particular system type is reached, and then a slower rise until biological or cultural evolution creates a new type. Chaisson’s narrative closely aligns with the arguments here, once it is seen that the energy rate density measures the stored energy flowing through the system per kg. This energy carries the programme bits that transform the system. In which case, the energy rate density can be converted to the “bit rate density” of the programmes that drives the system further from equilibrium.

When a system is maintained in a homeostatic state, the more degraded the energy that exits the system, the further the homeostatic state will be from equilibrium. Maximum ordering requires that the maximum number of programme bits be converted to heat bits to be ejected from the system. As is discussed further below, the greater the entropy production of the system the more ordered it is, whether entropy is measured in algorithmic terms or thermodynamic terms.

The next step here is to develop a narrative to explain how order is created in an evolutionary economy.

## 5. The Economic Narrative Behind an Economy as a Computational System

Replication processes, drive a system such as an economy to its carrying capacity. This is a highly ordered homeostatic state, far-from-equilibrium. The algorithmic description, as in Equation (1) is extremely short relative to a disorganised system of minerals and chemicals, as it describes a highly organised system. The drive for increasing order manifests itself in two different ways as is discussed next. The first is the drive to increase the carrying capacity corresponding to Lotka’s Maximum Power Principle (MPP) [47,48]. The second, while increasing the carrying capacity, also is a drive for energy efficiency leading to the Maximum Entropy Production Principle (MEPP).

### 5.1. MPP

At the carrying capacity, the system maximises its use of the exergy flowing into the system, corresponding to Lotka’s maximum power principle (MPP), which is in effect a “maximising power intake principle” (see discussion [49]). The simplest economy of hunter-gatherers will reproduce (replicate) until the carrying capacity is reached where the birth rate equals the death rate. If there are environmental changes, affecting energy access, the birth and death rate will adjust to maintain the system. The principle is a direct consequence of replication processes and selection. If two similar replicating systems, such as two hunter gatherer communities compete for the same resources, the one with the highest replicating efficiency and the lowest death rate will completely dominate the system as it maximises the utilisation of exergy increasing the order, analogous to the growth of self-replicating polynucleotides [20,50,51].

#### Innovation and Tools

When simple economic agents use their computational capacity to innovate and create tools, the carrying capacity increases. Wood can be burned to warm a community, while clothes and dwellings allow the community to survive over a wider area, increasing the order and the carrying capacity increases by maximising resource utilisation, as captured by the MPP. Similarly technological innovation can involve better farming practices, such as extracting resources from waste as fertiliser, irrigation and the use of animals to plough. Tools and machines, which access new computational pathways, amplify the capabilities of an agent, significantly increasing the order of the economy by driving it further from equilibrium, but usually at the cost of high resource use in fuels and greater waste expenditure. However, smarter technologies (such as computational technologies) can maintain or increase order using less energy and therefore generate less waste, and some technologies can use resources more efficiently to minimise waste. Hidalgo [13] sees the equivalent of this ordering process as crystallised imagination. He illustrates this with the idea of a medicinal pill. When swallowed, the pill is designed to activate real-world computations embedded within the human body to increase human survival.

Innovation that creates new technologies is an evolutionary process [29]. However, as learning allows other agents to use technological know-how, computational capability diffuse through the economy at very little cost, enabling the whole system to function at a higher level. The high quality of life evident in developed economies relies on tools and external energy to enhance the value of labour, but always with the proviso that higher entropy waste must also be ejected.

### 5.2. MEPP Further Increase in Order

While replication and selection processes drive the economic system towards MPP, another set of processes that interconnect different parts of the economy maximise the entropy production of the whole system. The ecological argument of Schneider and Kay [52] shows that a more complex ecology, formed by natural selection process, degrades the useful energy more effectively, and pumps out more useless energy at a greater rate than a simple ecology. Similarly, in a highly connected economy from a computational perspective, the waste from one component of the system still retains residual programme information that can become the resource input for another, extracting more value from the useful energy before being discarded [14]. The more highly connected and ordered the economy, and the more hierarchical its structure, the greater the entropy in the final waste output, whether it is measured in bits or in thermodynamic terms. This allows the evolutionary stages of an economy to be understood in terms of the Maximum Entropy Production Principle. Herrmann-Pillath [49] in Figure 2.15 shows the relationship between the Maximum Entropy Principle, MPP and MEP while the associated discussion identifies these principles as a manifestation of the Second Law of thermodynamics in non-equilibrium systems from a whole system perspective. Here these principles are derived from a bottom up process.

#### 5.2.1. Trade

From an algorithmic perspective, order is generated as programmes bits that enter one part of the economy pass to subroutines in another part of the economy. The simplest example is trade. Order increases if potatoes can be traded for carrots, rather than be left to rot, as more energy can be utilised for the same inputs. From an algorithmic point of view, trade is equivalent to sharing information between the routines associated with different agents. Fewer information bits embodied in the useful energy need to enter the system to define the more ordered system [20]. As trade increases the efficiency of resource use, the carrying capacity increases in the first instance. Trade drives the system further from equilibrium. One can conclude that, in a resource constrained environment interdependence through trade is the major alternative to conflict between agents.

#### 5.2.2. An Interconnected Economy

As Hidalgo [13] and Hidalgo and Hausmann [53] point out, individuals, teams and cities are pockets of information, knowledge and know-how. The economy is where people accumulate knowledge and know-how to create order. Large networks store a large amount of know-how and knowledge. The complex economy is richer because it has richer interconnections and is therefore more ordered than a simple hunter-gatherer economy. As economic complexity increases, agents develop mutually beneficial interconnections to form more ordered collective computational networks. Infrastructure, such as transport allows computational resources embodied in objects to be used elsewhere, but with an associated energy cost. Figure 1 illustrates how primitive agent structures can become interconnected. Firms, cities and nation states share informational resources horizontally and vertically between subsystems through trade and other relationships. This structural order is critical to a modern economy driving it further from equilibrium. The structural map of an ordered economy shows how routines, as know-how, can harness routines in the natural world to generate ordered productive structures. Cities pass information to other cities, or farmers provide food to cities, while within the cities, firms pass informational resources and goods embodying order to customers. The US adapted economically to its large geographical area, increasing scale and scope by harnessing coal as a cheap fuel, leading to the emergence of connected infrastructures, and giant companies [54]. Such structures are highly ordered using the available information resources more effectively, extracting the maximum benefit from these resources, while ejecting more disorder as bits in the degraded waste.

The argument here is not that such structures are inevitable but that when they occur, the order of the economic system increases, becoming further from local equilibrium. Selection processes are likely to favour agents who become more strategically connected as information resources are better used.

Initially, increased economic order corresponds to an increase in the carrying capacity. However, when sophisticated economic structures emerge and highly ordered artefacts and tools arise, order is crystallised in objects and structures rather, than just in the carrying capacity.

An economy never quite gets to its maximum entropy production state as residual energy in waste still exists that can be processed by the birds, rats and microbes that degrade the rubbish in tips. However, the total econ-biosystem seeks to maximise entropy production driving everything to equilibrium more rapidly than a much simpler system.

From a related perspective Hidalgo [13] developed the concept of economic complexity which is linked to computational know-how, arguing that more complex products depend on lager and more intricate networks, and that more successful economies trade in more diverse goods. Hidalgo [13] (p. 157) illustrates this by showing that Singapore has greater economic complexity than Chile or Pakistan in terms of the ubiquity of the Singaporean products and the diversity of its export countries.

Financial institutions are critical components of an interconnected economy. These institutions exchange information with other institutions and structures. Money behaves as a numerical code to signal the quantity of resources needed for the computational agents to implement routines.

## 6. Conclusions

In contrast to the neoclassical understanding of economic equilibrium where economic forces balance, once energy is considered it is seen that an economy is far from its local thermodynamic equilibrium. From the AIT perspective, an ordered economy, with the same basic constituents as a less ordered economy, is much simpler to describe on a reference UTM as seen by the order in a structural map representing the economy. An ordered economy can only be maintained distant from its local equilibrium by what can be seen as computational bits carried by the useful energy to regenerate the economy and also eject waste.

Human know-how can initiate further ordering processes through trade, through innovation by creating new technologies, and forming highly organised economic structures to add value. As more ordered economies better use their resources, they are more effective economies. The approach clarifies some of the issues raised by the Bioeconomic school [5,33,34,42], providing an overarching economic perspective that can integrate other economic perspectives. By drawing attention to the role of useful energy and evolutionary technological change and the need to eject waste, it clarifies the strategic choices to determine how best to improve societal well-being using limited natural resources.

## Figures and Tables

**Table 1:** Algorithmic order and structural order.

Algorithmic Order	Matching Characteristics of Structural Order
Information and Bits	Organisation and Resources
Short algorithmic description =Low algorithmic entropy =Ordered system.	Organised structure.
Nested and interconnected subroutines (Figure 1).	Nested and interconnected structures mapped by a simple flow diagram.
Fewer information bits describe system.	Fewer resources are needed.
Information bits pass between routines.	Resources flow between system components.
Disorder as bits ejected.	Waste as low grade energy is ejected as heat.

**Table 2:** Algorithms that map ordering through natural processes.

Algorithms that Implement Natural Processes	Natural Processes
Human routines call algorithms that drive natural processes.	Humans utilise concentrated energy as food or fuel.
Separating order from disorder.	Useful energy does work heating the system.
Waste containing heat bits ejected, leaving ordered bits behind.	Waste and heat ejected, leaving order behind.

## Appendix A. Reversibility Implications

As was discussed earlier, the traditional entropies apply to a macrostate, whereas the algorithmic entropy is for a specific microstate in macrostate, but where all microstates have the same algorithmic entropy. However the requirements for reversibility force the algorithmic approach to fully account for all bits, and therefore bit flows in and out of a system, such as an economy, completely identify with the corresponding thermodynamic entropy flows through Landauer’s principle.

### Appendix A.1. The Local Equilibrium

A state far-from-equilibrium has bits stored as available energy in the programme instructions that implement natural laws. Here arguments become simpler if the local equilibrium state is taken to be the set of states where all the stored energy in the programme instructions has been converted to heat, i.e., it is a strict definition of equilibrium, rather than one defining the most probable set of states, or one that also includes rare fluctuations from the strict equilibrium. In which case, at the local equilibrium, bits that were stored as programme instructions become completely dispersed as heat, and are unavailable to work within the system. Remaining structures are deemed inert in the short term as any stored energy is unavailable over the time frame of interest. Instructions to maintain reversibility still exist at the local equilibrium, but not as software instructions stored in available energy, but as the rules determining the kinetic interactions of species, analogous to the rules determining the behaviour of gates in a conventional computer.

While an irreversible UTM must contain an instruction to halt the programme once an equilibrium configuration is reached, the halt requirement for a reversible UTM is equivalent to the requirement that all available energy storing information becomes dispersed as heat bits. If a programme *p* embodied in a real-world UTM drives the system to an equilibrium microstate $e_{1}$ but does not halt, the number of bits to generate this microstate is |p|, as the energy in the programme bits specifying $e_{1}$, are converted to heat bits. However, the real-world programme does not halt as the residual instructions embodied in the kinetic states of the system maintain reversibility and drive $e_{1}$ to another equilibrium microstate $e_{2}$ having the same algorithmic entropy. Once this has happened, any fluctuations from equilibrium can be ignored and the system just shuffles thoroughly all the states with the same algorithmic entropy without the need to halt. There are $2^{\left|p\right|}$ possible microstates with the same algorithmic entropy in the set of equilibrium microstates. The Shannon entropy of this set is $log_{2}2^{\left|p\right|}$, i.e., $H_{sh}=\left|p\right|$. Consequently, the algorithmic entropy of the particular strict equilibrium microstate $=H\left(e_{1}\right)=\left|p\right|=H_{sh}$.

## Abbreviations

The following abbreviations are used in this manuscript:

AIT	Algorithmic Information Theory
UTM	Universal Turing Machine
AMSS	Algorithmic Minimum Sufficient Statistic
CPU	Central Processing Unit
MDPI	Multidisciplinary Digital Publishing Institute
